# Degradation Characteristics of Nicosulfuron in Water and Soil by MnO_2_ Nano-Immobilized Laccase

**DOI:** 10.3390/toxics12080619

**Published:** 2024-08-21

**Authors:** Wanlei Yue, Xin Wang, Jiale Zhang, Jia Bao, Mengqin Yao

**Affiliations:** 1 School of Environmental and Chemical Engineering, Shenyang University of Technology, Shenyang 110870, China; m15222487370@163.com (W.Y.); 18704120001@139.com (J.Z.); baojia@sut.edu.cn (J.B.); 2 School of Chemistry and Chemical Engineering, Guizhou University, Guiyang 550025, China; mqyao@gzu.edu.cn

**Keywords:** immobilized laccase, nicosulfuron degradation, MnO_2_ nanomaterials

## Abstract

As a typical sulfonylurea herbicide, nicosulfuron is mainly used to control grass weeds and some broadleaf weeds in corn fields. However, as the amount of use continues to increase, it accumulates in the environment and eventually becomes harmful to the ecosystem. In the present study, a new metallic nanomaterial, δ-MnO_2_, was prepared, which not only has a similar catalytic mechanism as laccase but also has a significant effect on pesticide degradation. Therefore, the bicatalytic property of MnO_2_ can be utilized to improve the remediation of nicosulfuron contamination. Firstly, MnO_2_ nanomaterials were prepared by controlling the hydrothermal reaction conditions, and immobilized laccase was prepared by the adsorption method. Next, we investigate the effects of different influencing factors on the effect of immobilized laccase, MnO_2_, and free laccase on the degradation of nicosulfuron in water and soil. In addition, we also analyze the metabolic pathway of nicosulfuron degradation in immobilized laccase and the bicatalytic mechanism of MnO_2_. The results demonstrated that the degradation rate of nicosulfuron in water by immobilized laccase was 88.7%, and the optimal conditions were 50 mg/L, 25 h, 50 °C, and pH 5. For nicosulfuron in soil, the optimal conditions for the degradation by immobilized laccase were found to be 151.1 mg/kg, 46 °C, and pH 5.9; under these conditions, a degradation rate of 90.1% was attained. The findings of this study provide a theoretical reference for the immobilized laccase treatment of sulfonylurea herbicide contamination in water and soil.

## 1. Introduction

Sulfonylurea herbicides are one of the mainstream herbicides in the world today, with a broad spectrum of herbicidal properties, and their main structures include aromatic rings, heterocycles, and sulfonylurea bridges [1]. It is considered a “new type of pesticide” because of its high efficiency, low toxicity, and high selectivity, and is widely used for weed control in maize, soybean, rice, wheat, rapeseed, and other crops [2,3,4]. In recent years, the production and application of sulfonylurea herbicides have developed more rapidly. Sales account for more than 11% of the global herbicide market [5]. Nicosulfuron, (2-[(4,6-dimethoxypyrimidin-2-ylcarbamoyl)sulfamoyl]-N,N-dimethylnicotinamide), one of the sulfonylurea herbicides, is the main herbicide used in the global maize cultivation process with its low usage, high herbicidal activity, and good crop selectivity [6,7,8]. Some studies have shown that nicosulfuron is commonly found in soil and groundwater, with residues of up to 2.3 g–4.7 g/acre in the soil and up to 3.29 μg/L in the groundwater [9,10]. In addition, its residual time in soil is strongly influenced by pH. In acidic soils, nicosulfuron is mostly susceptible to hydrolysis and photolysis, with a half-life of about 15–20 days, while in alkaline soils, the half-life can reach 190–250 days [11]. Nicosulfuron inhibits plant growth by inhibiting the activity of acetolactate synthase (ALS) [12]. Of the herbicides commonly used in maize fields, nicosulfuron has been reported to be the most likely to affect the growth of later crops [13]. Zhao et al. [8] found that beta vulgaris, as a back-crop of maize, had an inhibitory effect on the growth of beta vulgaris when nicosulfuron residues reached 0.3 μg/kg. Xu et al. [14] found that nicosulfuron significantly inhibited the growth of nicosulfuron-sensitive lines of maize. Environmental residues of nicosulfuron are not only toxic to a wide range of aquatic plants and disruptive to crop rotations, but they also have an impact on microbial populations in the soil [15]. Recent studies have confirmed that nicosulfuron also induces hypoglycemia in humans, increasing the risk of cardiovascular disease [11]. Therefore, research on how to remove residual nicosulfuron from water and soil has become an urgent global problem.

Biological methods have gradually become the main focus of herbicide degradation research due to their advantages of high efficiency, safety, and low cost. Many experiments have been conducted to successfully degrade nicosulfuron by biological methods [16,17,18]. Laccase has shown potential application value in degrading herbicides. However, free laccase has the disadvantages of poor stability, high pH and temperature requirements, and non-reusability [19]. Studies have shown that immobilized laccase effectively ameliorates the problems present with free laccase. Al-sareji et al. [20] immobilized laccase with date stone and found that the storage, pH, and thermal stability of immobilized laccase were improved. Sun et al. [21] immobilized laccase with mesoporous ZIF-8; they found that the stability and reusability of the immobilized laccase were significantly improved, and the removal of bisphenol A by the immobilized laccase was up to 90.28% even under adverse conditions.

Currently, few scholars have conducted dual studies on the metabolic pathway of nicosulfuron and the principle of action of immobilized laccase technology simultaneously. Therefore, in this paper, immobilized laccase (MnO_2_@Lac) was used to degrade nicosulfuron in water and soil samples. Based on a single-factor experiment, the degradation effects of immobilized laccase, nano-MnO_2,_ and free laccase on soil nicosulfuron were investigated by response surface methodology (RSM), and the degradation conditions were optimized. The synergistic mechanism of nanometer MnO_2_ and laccase was also explored, and the degradation pathways of nicosulfuron were analyzed, opening up a new way for the remediation of herbicides.

## 2. Materials and Methods

### 2.1. Materials

Laccase (EC 1.10.3.2) was purchased from Novozymes (China) Biotechnology Co., Ltd (Tianjin, China). Nicosulfuron(2-((4,6-dimethoxypyrimidin-2-ylcarbamoyl)sulfamoyl)-N,N-dimethylnicotinamide, purity > 97%) was purchased from Guangzhou Puxin Biotechnology Co., Ltd (Guangzhou, China).

### 2.2. Preparation and Characterization of Immobilized Laccase with MnO_2_ Nanoparticles

δ-MnO_2_ materials were prepared by the hydrothermal method [22]. MnSO_4_ was used as the manganese source and KMnO_4_ as the oxidizer. The mass ratio m(MnSO_4_): m(KMnO_4_) = 3:8 was added to deionized water and stirred. The homogeneously dispersed precursor solution was then transferred to a 200 mL reactor and kept at 100 °C for 3 h. After cooling to room temperature, the precipitate was filtered and rinsed until the filtrate was neutral. The δ-MnO_2_ was obtained by drying at 80 °C for 12 h.

Preparation of immobilized laccase: 10 mg of laccase (1.0 mg/mL) and 10 mg of δ-MnO_2_ nanomaterials were added to 10 mL of acetic acid-sodium acetate buffer, pH 5. The immobilized laccase was obtained by shaking at 30 °C for 4 h in a 150 rpm constant-temperature shaker and then dried by filtration. Scanning electron microscopy (SEM, Carl Zeiss AG, Oberkochen, BW, Germany) was used for microscopic morphology analysis of the δ-MnO_2_ nanomaterials, and immobilized laccase. Then, it was characterized by Fourier transform infrared spectroscopy (FT-IR, Thermo Nicolet Corporation, Madison, WI, USA).

### 2.3. Extraction Method and Determination of Degradation Rate of Nicosulfuron

The reaction substrate in this experiment was nicosulfuron, and the concentration of nicosulfuron was determined using an Agilent 1260 high-performance liquid chromatograph (Agilent Technologies Inc., Shanghai, China) during the analysis. The mobile phase consisted of 0.1% aqueous acetic acid and acetonitrile, and the column temperature was set to 30 °C; the detection wavelength was 241 nm; the flow rate was 1.0 mL/min; and the injection volume was 20 μL.

Three types of samples were prepared: a standard sample, a sample in water, and an experimental sample in soil. Nicosulfuron acetonitrile solution (200 mL) consisted of 2 mg of nicosulfuron and 10 mL of acetonitrile, which was used as a standard sample to be tested. A total of 2 mg of nicosulfuron, 10 mL of acetonitrile, 10 mL of MnO_2_@Lac solution and 100 μL of 0.1% acetic acid were mixed and shaken on a shaker at 220 r/min 25 °C for 30 min. For the experimental samples in soil, 2 mg of nicosulfuron was added to 10.75 mL of acetonitrile, which was sprayed uniformly in 5 g of autoclaved soil. Subsequent experimental procedures were the same as for the samples in water. All the above samples to be tested were taken in 1 mL, passed through a 0.22 μm filter membrane, and left to be tested. The recoveries of nicosulfuron in water and soil were calculated to be 78.65% and 72.65%, respectively, which proved that the extraction technique could meet the experimental requirements.

### 2.4. Optimization of Degradation Conditions for Nicosulfuron in Water

The degradation was carried out on aqueous solutions of nicosulfuron at medium to high concentrations. The degradation effects of immobilized laccase, MnO_2_, and free laccase on nicosulfuron were determined separately, and the experiments are described below 1 mg MnO_2_@Lac, 1 mg MnO_2,_ and 1 mg free laccase were added to 50 mg/L nicosulfuron solution at pH 5, respectively, and the content of nicosulfuron was detected after reacting for a certain time at 30 °C on a shaker at 160 r/min. To determine the effects of temperature, pH, initial nicosulfuron concentration, and reaction time on the degradation of nicosulfuron, the temperatures were set at 20, 30, 40, 50, 60, and 70 °C; pH = 3, 4, 5, 6, 7, and 8; the initial nicosulfuron concentrations were 10, 30, 50, 80, 100, 150, and 200 mg/L; and the reaction times were 5, 10, 15, 20, 25, 30, 35, 40, and a single-factor experiment was conducted to further optimize the degradation conditions of nicosulfuron.

### 2.5. Optimization of Degradation Conditions for Nicosulfuron in Soil

RSM was used to optimize the degradation conditions of immobilized laccase for nicosulfuron in soil. Three factors were selected that had a significant effect on the degradation rate of nicosulfuron: pH (A), temperature (B), and initial nicosulfuron concentration (C). The response values included the degradation rates of immobilized laccase, MnO_2_, and free laccase on nicosulfuron (Table 1).

### 2.6. Data Analysis

All experimental treatments were repeated three times. Charts were generated using Origin 2019b (Origin Lab Co., Northampton, MA, USA) and regression model analysis of variance with Design-Expert 12 (Stat-Ease Inc., Minneapolis, MN, USA). All data were presented as mean values, with the standard deviation represented by error bars.

## 3. Results and Discussion

### 3.1. Preparation and Morphological Characterization of MnO_2_ Nanomaterials and Immobilized Laccase

Hydrothermal methods are widely used in the manufacture of new materials with unique structures and shapes due to their high purity, good crystallinity, and multiple morphologies. In the process of hydrothermal material synthesis, the regulation of key parameters is crucial. The process of hydrothermal synthesis of materials involves several core parameters, which include the type and addition ratio of reactants, the time and temperature of the reaction, as well as the acidity and alkalinity, which enable the synthesis of MnO_2_ with various morphologies. KMnO_4_ and (NH_4_)_2_S_2_O_8_ are strong oxidizing agents, and MnSO_4_·H_2_O acts as a reducing agent, and a typical redox reaction occurs when preparing the samples as follows:

3MnSO_4_ + KMnO_4_ + 2H_2_O → 5MnO_2_ + K_2_SO_4_ + 2H_2_SO_4_ (1)

MnSO_4_ + (NH_4_)_2_S_2_O_8_ → MnO_2_ + (NH_4_)_2_SO_4_ + Mn_x_O_y_ (2)

The reaction Equation (1) contains two half-reactions, and the equation is shown below:

Mn^2+^ + 2H_2_O → MnO_2_ + 4H^+^ + 2e^−^ (E^0^ = 1.23 eV) (3)

MnO_4_^−^ + 4H^+^ + 3e^−^ → MnO_2_ + 2H_2_O (E^0^ = 1.23 eV) (4)

Based on the value of E^0^, the Gibbs free energy ΔG_0_ of the reaction in Equation (1) can be estimated to be −269.2 kJ/mol, indicating a strong tendency for the completion of the reaction. In addition, the temperature plays an important role in the potentials of MnO_4_^−^/MnO_2_ and MnO_2_/Mn^2+^. Therefore, the reaction rate of hydrothermal synthesis can be varied, resulting in the formation of MnO_2_ with different sizes and morphologies [23].

**Figure 1:**
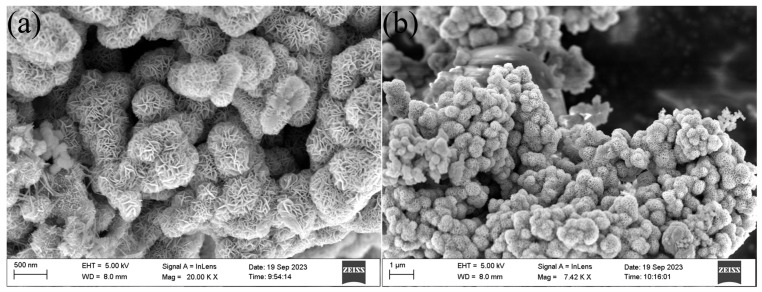
SEM images of δ-MnO_2_ (**a**) and MnO_2_@Lac (**b**).

SEM scans of δ-MnO_2_ material and MnO_2_@Lac are shown in Figure 1. Figure 1a shows the SEM image of δ-MnO_2_. It consists of nanosheets with a thickness of about 20 nm. These nanosheets grow vertically from the center and gradually form flower-like nanorods with a diameter of about 4 μm. Figure 1b shows that the MnO_2_@Lac surface can be seen in the form of flower ball-like MnO_2_ as well as spherical laccase attached to the surface of MnO_2_. The increase in the specific surface area caused by the wrinkles on the surface of MnO_2_ facilitates the attachment of laccase, and the morphology of MnO_2_ did not change before or after the immobilization.

**Figure 2:**
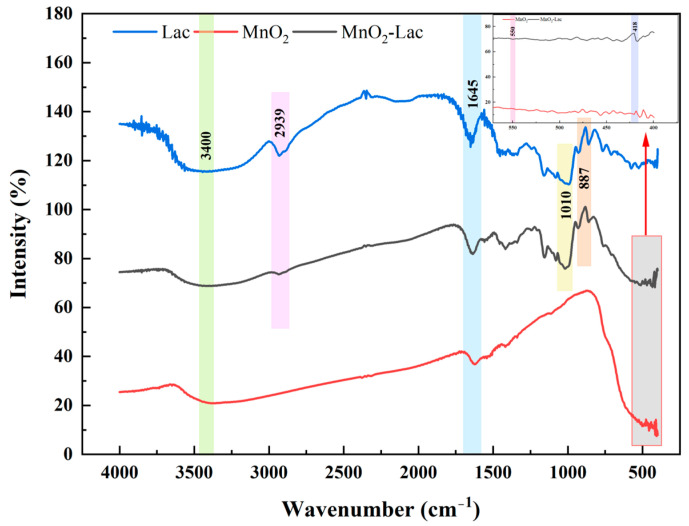
FT-IR images of free laccase, MnO_2_, and MnO_2_@Lac.

Figure 2 shows the FT-IR of free laccase, MnO_2_@Lac and MnO_2_. Two peaks were observed at 887 cm^−1^ and 1010 cm^−1^, corresponding to the C-N bond stretching vibration and C-O bond stretching vibration of laccase, respectively. The characteristic peak at 2939 cm^−1^ is attributed to the antisymmetric vibration of the methylene group [24,25,26]. The spectral lines of MnO_2_ and MnO_2_@Lac show characteristic peaks at 550 cm^−1^ and 418 cm^−1^. They correspond to the stretching vibrations of Mn-O bond (550 cm^−1^) and Mn-O-Mn bond (418 cm^−1^), respectively [27]. Thus, it is shown that laccase has been immobilized into the material. However, the characteristic peaks at 3400 cm^−1^ and 1645 cm^−1^ are attributed to the O-H stretching vibration and the H-O-H bending vibration of water, respectively [28].

### 3.2. Degradation of Nicosulfuron in Water by Immobilized Laccase

**Figure 3:**
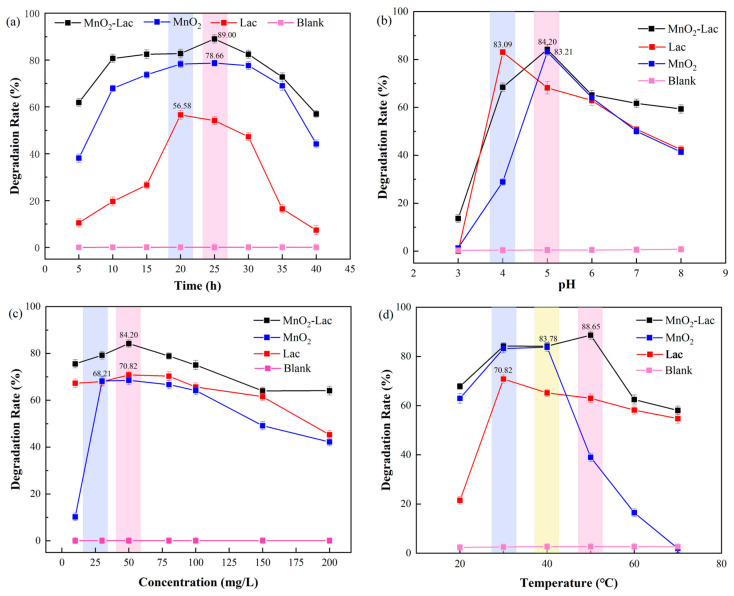
Optimization of degradation conditions for nicosulfuron in water: reaction time (**a**); pH (**b**); initial concentration of nicosulfuron (**c**); and temperature (**d**).

The effects of different reaction times (5–40 h), pH (3–8), initial pesticide concentrations (10–200 mg/L), and temperatures (20–70 °C) on the degradation rate of nicosulfuron were investigated using a single-factor experiment. As can be seen in Figure 3a, the degradation of nicosulfuron by laccase after immobilization was significantly enhanced. The degradation rate of nicosulfuron by MnO_2_@Lac reached the highest 89.0% at 25 h. Overall trend in nicosulfuron degradation: MnO_2_@Lac > MnO_2_ > free laccase. This may be due to the ability of MnO_2_ to mimic the catalytic oxidation of laccase, and laccase synergistic degradation of nicosulfuron. In addition to the large number of hydroxyl groups in MnO_2_, nicosulfuron has a certain degree of adsorption and oxidation removal effect [29]. For the effect of pH on the degradation rate of nicosulfuron (Figure 3b), the optimum pH for MnO_2_@Lac was 5, which resulted in a degradation rate of 84.2%. At pH3, the degradation rate of nicosulfuron by free laccase and MnO_2_ was almost zero, while the MnO_2_@Lac still had a degradation rate of 13.6%. This demonstrates that immobilized laccase has a wider pH application range than free laccase and MnO_2_. Figure 3c shows that the effect of initial pesticide concentration on the degradation rate shows an overall trend of increasing and then decreasing. When the initial concentration of nicosulfuron was 50 mg/L, the maximum degradation rate was 84.2%, and then the degradation rate began to decrease. When the concentration of nicosulfuron exceeded 50 mg/L, the toxicity of the high concentration of pollutants gradually appeared, resulting in the inactivation of some laccase enzymes and a decrease in degradation efficiency [26]. Temperature has two main effects on enzyme-catalyzed reactions: first, as the temperature rises, the diffusion of reactants and substrates becomes faster, which helps to improve the efficiency of the enzyme-catalyzed reaction; second, laccase is a class of polyphenol oxidases whose activity is susceptible to changes in temperature and which may be rendered inactive once the temperature is too high [30,31]. As shown in Figure 3d, the optimum temperature of MnO_2_@Lac is 50 °C, when the degradation rate is 88.7%. The optimum temperatures of free laccase and MnO_2_ were 30 °C and 40 °C, respectively, and the maximum degradation rates were 70.8% and 83.8%, respectively. The degradation rate of nicosulfuron by MnO_2_@Lac was minimally affected by temperature compared with free laccase and MnO_2_ and was higher than 50.0% in the temperature range of 20 °C~70 °C. In conclusion, the optimal degradation conditions for the degradation of nicosulfuron in water by MnO_2_@Lac were the reaction time of 25 h, pH 5, the initial pesticide concentration of 50 mg/L, and the temperature of 50 °C.

### 3.3. Reusability and Enzymatic Degradation Kinetics of Nicosulfuron Degradation by Immobilized Laccase Enzymes

**Figure 4:**
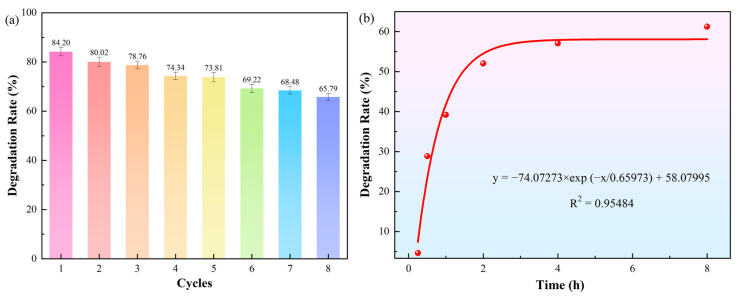
Reusability of immobilized laccase for degradation rate of nicosulfuron (**a**) and kinetics of enzymatic degradation of nicosulfuron (**b**).

To test the reusability of immobilized laccase for the degradation of nicosulfuron, MnO_2_@Lac was recovered and reused for the degradation of nicosulfuron, and the experimental procedure was repeated eight times. After 5 cycles, the degradation rate of nicosulfuron by MnO_2_@Lac slightly decreased from 84.2% to below 70.0% (Figure 4a). This may be due to the gradual depletion of MnO_2_@Lac with the increase in the number of cycles, which affected the overall oxidizing performance. Secondly, this may be due to the shedding of laccase during multiple rinsing processes [32]. However, after 8 cycles, the degradation rate of nicosulfuron could still be maintained at 65.0% or higher, which demonstrated the high reusability of the immobilized laccase and reduced the cost of the enzyme while ensuring a higher degradation rate, which provided the possibility of high-volume use of the immobilized laccase. Meanwhile, the enzymatic degradation kinetics of nicosulfuron was determined in this paper (Figure 4b). The half-life of 50 mg/L nicosulfuron was 1.46 h in the first-level kinetic model fitting.

### 3.4. Studies on the Degradation of Nicosulfuron in Soil by Immobilized Laccase

According to the results in Section 3.2, the highest degradation rate of nicosulfuron in the water body could reach 84.2%, and the temperature, pH, and initial concentration of pesticide had a large effect on the degradation rate of nicosulfuron. To improve the experimental efficiency and shorten the optimization time, the RSM was used to analyze the optimum conditions for the degradation of nicosulfuron in soil by MnO_2_@Lac, MnO_2_, and free laccase.

#### 3.4.1. Regression Modeling Analysis of RSM 

Three factors (pH (A), temperature (B), and initial concentration of nicosulfuron (C)) were analyzed by regression fitting and regression modeling. The quadratic multiple regression equation obtained is as follows.

Y1 = −178.38125 + 39.23163 A + 3.18955 B + 1.05619 C − 3.26581 A^2^ − 0.034214 B^2^ − 0.003204 C^2^

Y2 = −69.43250 + 21.30625 A + 3.19292 + 0.166425 C − 1.68 A^2^ − 0.039122 B^2^ − 0.000521 C^2^

Y3 = −134.48000 + 33.80350 A + 2.84463 B + 0.721740 C − 2.82612 A^2^ − 0.029931 B^2^ − 0.002156 C^2^

The statistical analysis and model fitting data are shown in Table 2. For all three factors, only the quadratic term of the 2FI model presents significance as their *p*-values are less than 0.05. The lack-of-fit *p*-value is used as an additional indicator to assess the quality of the model. A higher lack of fit *p*-values indicates a strong fit. The model is considered to have a good fit when the loss-of-fit *p*-value exceeds 0.1 [33]. They all have *p*-values greater than 0.1 in the quadratic model and are therefore considered to have a good fit. R^2^ is the percentage change in the independent variable and its interacting response variable [34]. In addition, the difference between the adjusted R^2^ and the predicted R^2^ should remain within 0.2. This indicates that it does not contain a large number of statistically insignificant factors and that the model is reliable. Therefore, the quadratic model was chosen as the model for implementation.

The results of the analysis of variance using the Box–Behnken design of experimental protocol are shown in Table 3. For immobilized laccase, *p* < 0.0001, and for the lack of fit *p* = 0.8088 > 0.1. This indicates that the regression model fits well with actual experiments and is suitable for analyzing and predicting the conditions of MnO_2_@Lac degradation of nicosulfuron. The *p*-values of the three factors were pH (*p* = 0.0239), temperature (*p* = 0.0613), and initial concentration of nicosulfuron (*p* = 0.6610), where the *p*-value of pH was <0.05, which indicated that the effect of this factor was significant. Thus, it can be inferred that the degree of influence of the three factors on the rate of pesticide degradation was A > B > C. Similarly, for free laccase, *p* < 0.0001 for the overall model indicates that the regression model fits well with the actual experiment and is suitable for analyzing and predicting the conditions of nicosulfuron degradation by laccase. The *p*-values of the three factors were pH (*p* = 0.0042), temperature (*p* = 0.0470), and initial concentration of nicosulfuron (*p* = 0.4817). It can be inferred that the extent of their influence on the degradation rate: A > B > C. As can be seen from Table 3, the regression model for MnO_2_ is similar to the two models mentioned above, with the same analytical results, and pH is still the most significantly affected factor.

#### 3.4.2. Response Surface and Contour Analysis

**Figure 5:**
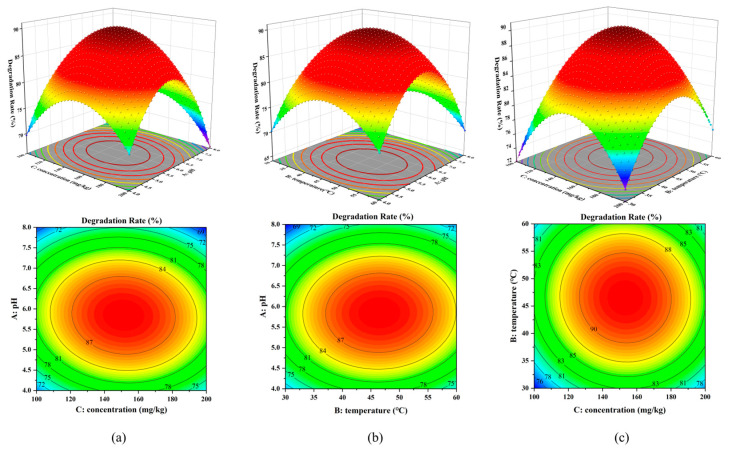
Response surface diagram (top) and contour diagram (bottom) of MnO_2_@Lac: interaction between pH and initial concentration of nicosulfuron (**a**), interaction between pH and temperature (**b**), and interaction between temperature and initial concentration of nicosulfuron (**c**).

To represent the interactions between the influencing factors more intuitively, response surface and contour maps of the interaction of the factors were plotted. In a response surface plot, the response value has a maximum if the opening of the surface is facing down. The more curved the curve and the faster the color change, the more significant the effect of the factors on the results [35,36]. In contour plots, if the contours are elliptical, the interaction between the factors has a significant effect on the response values. Figure 5 shows that the openings of the response surface plots are all downward, and the degradation rate shows a trend of increasing and then decreasing, indicating that there is a maximum in the response value. The steeper surface along the A-axis of the degradation rate relative to C in Figure 5a. This indicates that the effect of A on the degradation rate is more significant. This is consistent with the results obtained from the model developed in this paper. The contour plots in Figure 5a,b show an elliptical shape, indicating that the interaction terms AB and AC interact with each other to have a significant effect on the response values. In contrast, the contour graphs in Figure 5c appear nearly circular, indicating that the interaction of BC has a weak effect on the response values.

**Figure 6:**
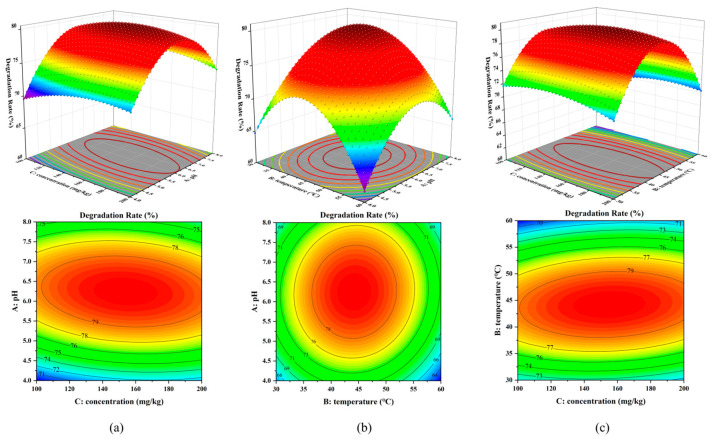
Response surface diagram (top) and contour diagram (bottom) of free laccase: interaction between pH and initial concentration of nicosulfuron (**a**), interaction between pH and temperature (**b**), and interaction between temperature and initial concentration of nicosulfuron (**c**).

Figure 6 shows the effect of three factors on the degradation rate of free laccase degradation of nicosulfuron. The response surface plots are all open downward, proving that there is a maximum in the degradation rate for all three sets of interaction experiments. Figure 6a shows that the degradation rate increases significantly with increasing A, but the degradation rate starts to decrease when A reaches about six. This is because the optimal pH of free laccase is around 4~5. As the pH increases, the enzyme activity gradually decreases or even inactivates, resulting in a lower degradation rate [37,38]. The steeper surface along the B-axis of the degradation rate relative to C in Figure 6c indicates that factor B has a more significant effect on the degradation rate. In the contour plots, the graphs in Figure 5a and Figure 6b are oval in shape and change color more rapidly, indicating that the interaction between AC and BC has a more significant effect on the response values. The graph in Figure 5b is rounded, indicating that there is no interaction between A and B.

**Figure 7:**
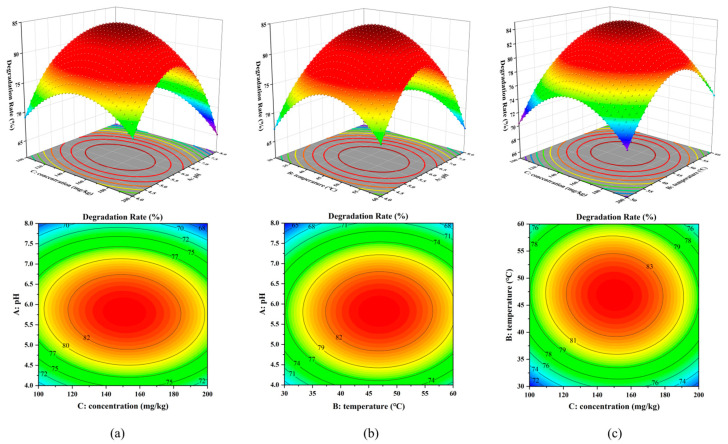
Response surface diagram (top) and contour diagram (bottom) of MnO_2_: interaction between pH and initial concentration of nicosulfuron (**a**), interaction between pH and temperature (**b**), and interaction between temperature and initial concentration of nicosulfuron (**c**).

The effects of the three factors on the degradation rate of nicosulfuron degradation by MnO_2_ are shown in Figure 7. The surface along the A-axis is steeper (Figure 7a,b), indicating that the effect of factor A on the degradation rate is more significant. Observation of their contour plots reveals that the graphs in Figure 7a,c are elliptical with faster color change. Therefore, it indicates that the interaction of AC and BC has a greater effect on the degradation rate of MnO_2_ degradation of nicosulfuron.

#### 3.4.3. Optimization and Analysis of Results

Numerical optimization was performed using Design Expert software. In this optimization process, the aim is to maximize the degradation rate. The optimization results of the software are shown in Table 4. The degradation of nicosulfuron fell within the 95% confidence interval. Therefore, these models are valid and accurately describe the interactions between the variables.

It is clear from Table 4 that MnO_2_@Lac degraded 90.1% of nicosulfuron in soil. According to previous reports, Zhou et al. [16] utilized the strain Oceanisphaera psychrotolerans LAM-WHM-ZC to degrade nicosulfuron in soil with a maximum degradation rate of 78.6%. Kang et al. [39] treated nicosulfuron using a nicosulfuron degrading enzyme produced in Bacillus subtilis strain YB1 and obtained a maximum degradation rate of 66%. In contrast, MnO_2_@Lac showed a greater degradation rate of nicosulfuron in soil.

### 3.5. Synergistic Mechanism of MnO_2_ Nanoparticles

Based on the results obtained in Section 3.4, comparing the degradation rates of MnO_2_@Lac, Lac, and MnO_2_ on nicosulfuron in soil, it can be concluded that the degradation effect is MnO_2_@Lac > MnO_2_ > free laccase. The reason for this phenomenon is that, on the one hand, the laccase molecules in the immobilized laccase are protected by the carrier. They can maintain higher enzyme activity in a wider temperature and pH range [20,37,40], and improve the degradation rate of pesticides. On the other hand, the synergistic catalytic effect of MnO_2_ materials.

**Figure 8:**
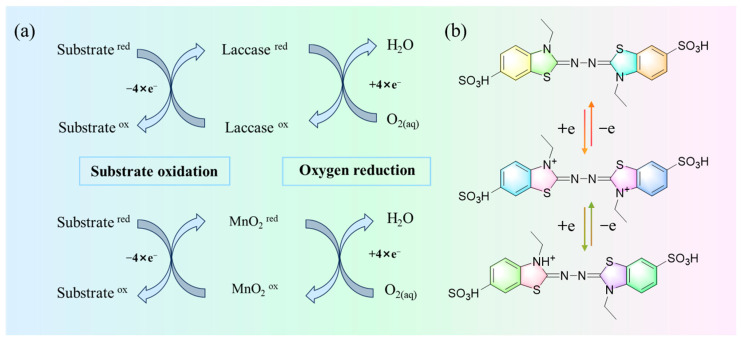
Mechanism diagram of substrate transformation between laccase and MnO_2_ (**a**) and ABTS electron transfer mechanism diagram (**b**).

MnO_2_ has the same substrate of action as laccase, 2,2′-azino-bis (3-ethylbenzothiazoline-6-sulfonic acid) (ABTS). ABTS is a special substrate for laccase and one of the most widely used mediators. As a mediator, ABTS plays the role of electron transfer between laccase and substrate (Figure 8b), which ultimately leads to substrate degradation [41]. Figure 8a shows the mechanism of substrate conversion by MnO_2_ and laccase. Certain manganese oxides (MnO_x_) can oxidize substrates by single electron transfer, and in turn, the reduced manganese oxides MnO_x_^red^ can be reoxidized to MnO_x_ by dissolved oxygen and reduced to water under certain conditions [42,43]. Wu et al. [44]. prepared and validated that two-dimensional ultrathin MnO_2_ nanofilm (Mn-uNF) can be used as a laccase mimic to degrade pollutants, and Mn-uNF possesses better stability than free laccase. In conclusion, MnO_2_ was able to synergize with laccase to degrade the target pollutants due to its laccase-like catalytic activity. Thus, the degradation rate of nicosulfuron by MnO_2_@Lac was significantly enhanced.

### 3.6. Analysis of Nicosulfuron Degradation Pathway

**Figure 9:**
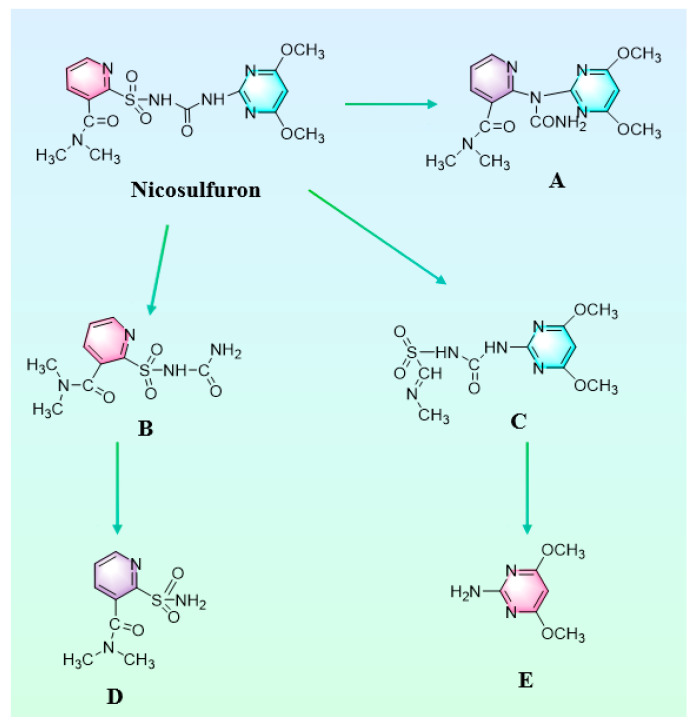
Degradation pathway of nicosulfuron in soil (A: 2-(1-(4,6-dimethoxy-pyrimidin-2-)-ureido)-N, N-dimethyl-nicotinamide; B: 2-aminosulfonyl-N, N-dimethylnicotinamide; C: N-(4,6-dimethoxypyrimidin-2-ylcarbamoyl)-1-(methylimino)methanesulfonamide; D: N, N-dimethyl-2-aminosulfonyl-isonicotinamide; E: 2-ylamine-4,6-dimethoxypyrimidinyl).

Sulfonylureas are weakly acidic herbicides, with pKa values generally ranging from 3 to 5. In aqueous solutions, herbicides exist mainly in the neutral state at pH values below pKa, while at pH values above pKa, they appear mainly in the anionic form. The main reason for the production of nicosulfuron degradation products is the breakage of the C-N, C-S, and S-N bonds in the sulfonylurea bridge [18]. Cleavage of the C-S bond of the sulfonylurea bridge produces 2-(1-(4,6-dimethoxy-pyrimidin-2-)-ureido)-N, N-dimethyl-nicotinamide (A). Breakage of the C-N bond of the sulfonylurea bridge produced 2-aminosulfonyl-N, N-dimethylnicotinamide (B), which was further deaminated by intermediate C to produce N, N-dimethyl-2-aminosulfonyl-isonicotinamide (D). Product C (N-(4,6-dimethoxypyrimidin-2-ylcarbamoyl)-1-(methylimino)methanesulfonamide) is generated due to the ring opening of the pyridine ring, which is followed by a condensation reaction, deamidation, and partial oxidation of the methoxy group to produce product E (2-ylamine-4,6-dimethoxypyrimidinyl) [45,46,47]. Summarizing the metabolite speculations, the metabolic pathway of nicosulfuron in soil degraded by MnO_2_@Lac is shown in Figure 9.

## 4. Conclusions

In this paper, the degradation of nicosulfuron in water and soil by MnO_2_@Lac, nano-MnO_2_, and free laccase was investigated by RSM, and the degradation conditions were optimized. The optimal degradation conditions of nicosulfuron in water by MnO_2_@Lac were 50 mg/L, 25 h, 50 °C, and pH 5. Under these conditions, the degradation of nicosulfuron in water reached 88.7% with a half-life of 1.46 h. The optimum conditions for the degradation of nicosulfuron in soil by immobilized laccase were 151.1 mg/kg, 46.5 °C, and pH 5.9. The degradation rate of nicosulfuron could reach 90.1%. We also used δ-MnO_2_ as a carrier to immobilize laccase. The laccase-like catalytic effect of nano-MnO_2_ was utilized to enable laccase and δ-MnO_2_ to synergistically degrade nicosulfuron, which led to the remarkable effect of MnO_2_@Lac in degrading residual nicosulfuron in water and soil samples. Thus, immobilized laccase can be applied in the remediation of soil contaminated with sulfonylurea herbicides and can be further experimented with in other types of herbicides. At the same time, this study provides a theoretical basis for the application of the removal of pesticide contaminants using mimetic enzyme nanomaterials in combination with bio-enzymatic methods.

## Figures and Tables

**Table 1:** Variables and levels in central composite design.

Factor	Level		
	−1	0	1
A-pH	4	6	8
B-Temperature (°C)	30	45	60
C-Initial pesticide concentration (mg/L)	100	150	200

**Table 2:** Statistical analysis and model fitting for RSM.

Statistical Test	Response	Source	Sum of Squares	df	Mean Square	F-Value	*p*-Value	Remark
Sequential model fitting	MnO_2_@Lac	Mean vs. Total	99,629.06	1	99,629.06			
		Linear vs. Mean	52.48	3	17.49	0.1619	0.9201	
		2FI vs. Linear	5.79	3	1.93	0.0138	0.9976	
		Quadratic vs. 2FI	1371.12	3	457.04	116.92	<0.0001	Suggested
		Cubic vs. Quadratic	5.36	3	1.79	0.3247	0.8088	Aliased
		Residual	22.00	4	5.50			
	Laccase	Mean vs. Total	89,189.58	1	89,189.58			
		Linear vs. Mean	31.80	3	10.60	0.2379	0.8683	
		2FI vs. Linear	5.96	3	1.99	0.0346	0.9908	
		Quadratic vs. 2FI	563.96	3	187.99	140.59	<0.0001	Suggested
		Cubic vs. Quadratic	1.32	3	0.4408	0.2194	0.8785	Aliased
		Residual	8.04	4	2.01			
	MnO_2_	Mean vs. Total	92,185.50	1	92,185.50			
		Linear vs. Mean	56.74	3	18.91	0.2496	0.8602	
		2FI vs. Linear	3.44	3	1.15	0.0117	0.9982	
		Quadratic vs. 2FI	937.92	3	312.64	50.06	<0.0001	Suggested
		Cubic vs. Quadratic	15.31	3	5.10	0.7184	0.5909	Aliased
		Residual	28.41	4	7.10			
Lack of Fit test	MnO_2_@Lac	Linear	1382.27	9	153.59	27.92	0.0029	
		2FI	1376.48	6	229.41	41.70	0.0015	
		Quadratic	5.36	3	1.79	0.3247	0.8088	Suggested
		Cubic	0.0000	0				Aliased
		Pure Error	22.00	4	5.50			
	Laccase	Linear	571.24	9	63.47	31.59	0.0023	
		2FI	565.29	6	94.21	46.89	0.0012	
		Quadratic	1.32	3	0.4408	0.2194	0.8785	Suggested
		Cubic	0.0000	0				Aliased
		Pure Error	8.04	4	2.01			
	MnO_2_	Linear	956.67	9	106.30	14.97	0.0096	
		2FI	953.23	6	158.87	22.37	0.0048	
		Quadratic	15.31	3	5.10	0.7184	0.5909	Suggested
		Cubic	0.0000	0				Aliased
		Pure Error	28.41	4	7.10			
**Model summary statistics**	**Response**	**Source**	**Std. dev.**	**R^2^**	**Adj. R^2^**	**Pred. R^2^**	**Press**	**Remark**
	MnO_2_@Lac	Linear	10.39	0.0360	−0.1864	−0.3965	2034.32	
		2FI	11.83	0.0400	−0.5360	−1.3492	3422.18	
		Quadratic	1.98	0.9812	0.9571	0.9175	120.11	Suggested
		Cubic	2.35	0.9849	0.9396		*	Aliased
	Laccase	Linear	6.68	0.0520	−0.1667	−0.4770	902.56	
		2FI	7.57	0.0618	−0.5011	−1.7113	1656.82	
		Quadratic	1.16	0.9847	0.9650	0.9448	33.72	Suggested
		Cubic	1.42	0.9868	0.9474		*	Aliased
	MnO_2_	Linear	8.70	0.0545	−0.1637	−0.3904	1448.51	
		2FI	9.91	0.0578	−0.5076	−1.3930	2493.05	
		Quadratic	2.50	0.9580	0.9041	0.7223	289.28	Suggested
		Cubic	2.66	0.9727	0.8909		*	Aliased

**Table 3:** Analysis of variance of regression model for response surface design.

Response	Source	Sum of Squares	Degree of Freedom	Mean Square	F Value	*p*-Value
MnO_2_@Lac	Model	1429.39	9	158.82	40.63	<0.0001
	Residual	27.36	7	3.91	-	-
	Lack of fit	5.36	3	1.79	0.32	0.8088
	Pure Error	22.00	4	5.50	-	-
	Cor Total	1456.75	16	-	-	-
Laccase	Model	601.73	9	66.86	50.00	<0.0001
	Residual	9.36	7	1.34	-	-
	Lack of fit	1.32	3	0.44	0.22	0.8785
	Pure Error	8.04	4	2.01	-	-
	Cor Total	611.09	16	-	-	-
MnO_2_	Model	998.10	9	110.90	17.76	0.0005
	Residual	43.71	7	6.24	-	-
	Lack of fit	15.31	3	5.10	0.72	0.5909
	Pure Error	28.41	4	7.10	-	-
	Cor Total	1041.82	16	-	-	-

**Table 4:** The proposed optimum conditions by RSM and experimental results.

Response	Optimum Conditions			Predicted Mean (%)	Experimental Results (%)
	pH	Temperature (°C)	Concentration (mg/kg)		
MnO_2_@Lac	5.9	46.5	151.1	90.3	90.1
Laccase	6.2	44.3	153.3	80.5	79.8
MnO_2_	5.8	46.8	150.7	84.9	84.2

## Data Availability

The data presented in this study are available on request from the corresponding author.
